# Supplementary material for: Structure of a Wbl protein and implications for NO sensing by M. tuberculosis
Source: Nat Commun. 2017 Dec 22;8:2280. doi: 10.1038/s41467-017-02418-y (PMC5741622; doi:10.1038/s41467-017-02418-y)
Supplement: Supplementary file 1 — Supplementary Information [file 41467_2017_2418_MOESM1_ESM.pdf]

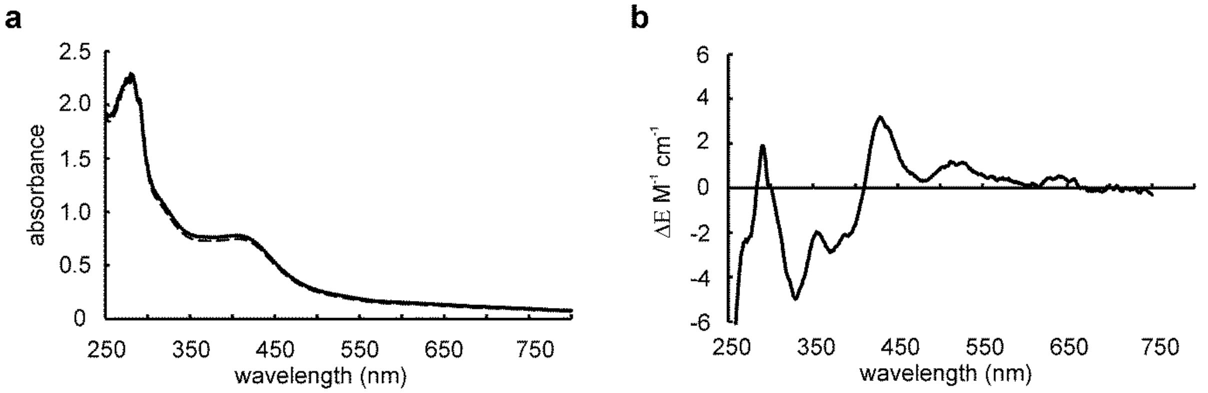

Supplementary Figure 1 | *Mycobacterium tuberculosis* WhiB1 expressed in *Mycobacterium smegmatis* possesses an O_2-stable [4Fe-4S] cluster. a, Absorbance spectra of WhiB1 isolated from recombinant *M. smegmatis* under aerobic conditions. Absorbance spectra obtained immediately after isolation (solid line) and 15 h later (broken line) for protein (42 μM cluster) in 50 mM Tris (pH 7.4) buffer containing 0.5 M NaCl, under aerobic conditions. **b**, CD spectrum of WhiB1 (30 μM cluster) in 10 mM NaH_2PO_4 (pH 7.4) buffer, containing 0.1 M NaCl.

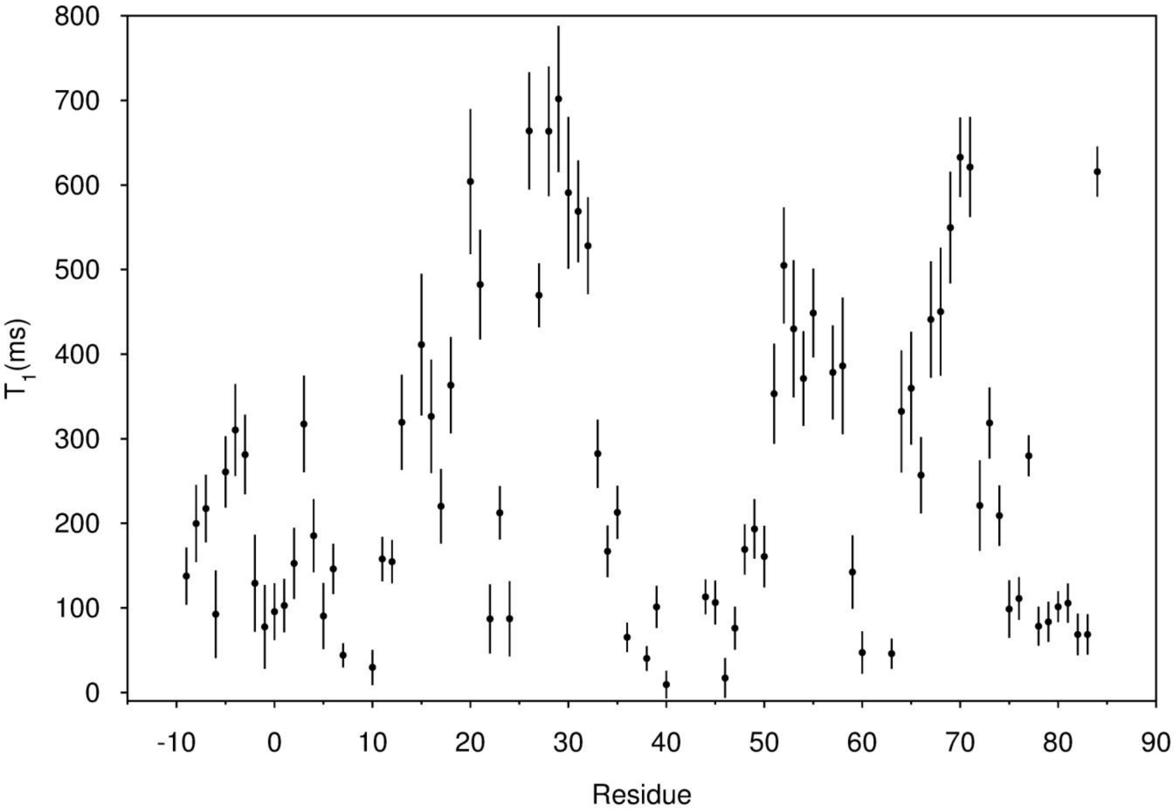

Supplementary Figure 2 | T_1 relaxation times of amide protons for WhiB1. Observed WhiB1 amide proton relaxation times plotted against amino acid residue number. WhiB1 initiating Met is designated as residue 1; negative residue numbers are amino acids in the His-TEV-tag. The error bars show the 95% confidence interval for T_1.

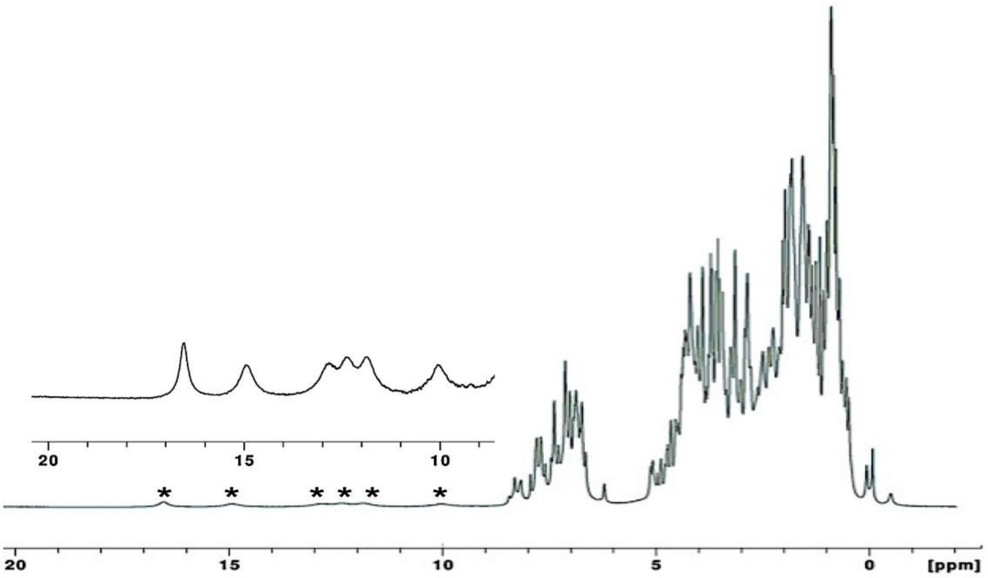

Supplementary Figure 3 | 1D ^{1}H-NMR spectrum of unlabeled WhIB1. WhiB1 (0.25 mM) in 25 mM NaH_2PO_4 (pH 7.0) containing 0.25 M NaCl. 1D ^{1}H-NMR spectra were recorded in the presence of 100% D_2O at 800 MHz. Peaks likely to represent iron-sulfur cluster ligands (cysteine Hβ resonances) are indicated (*).

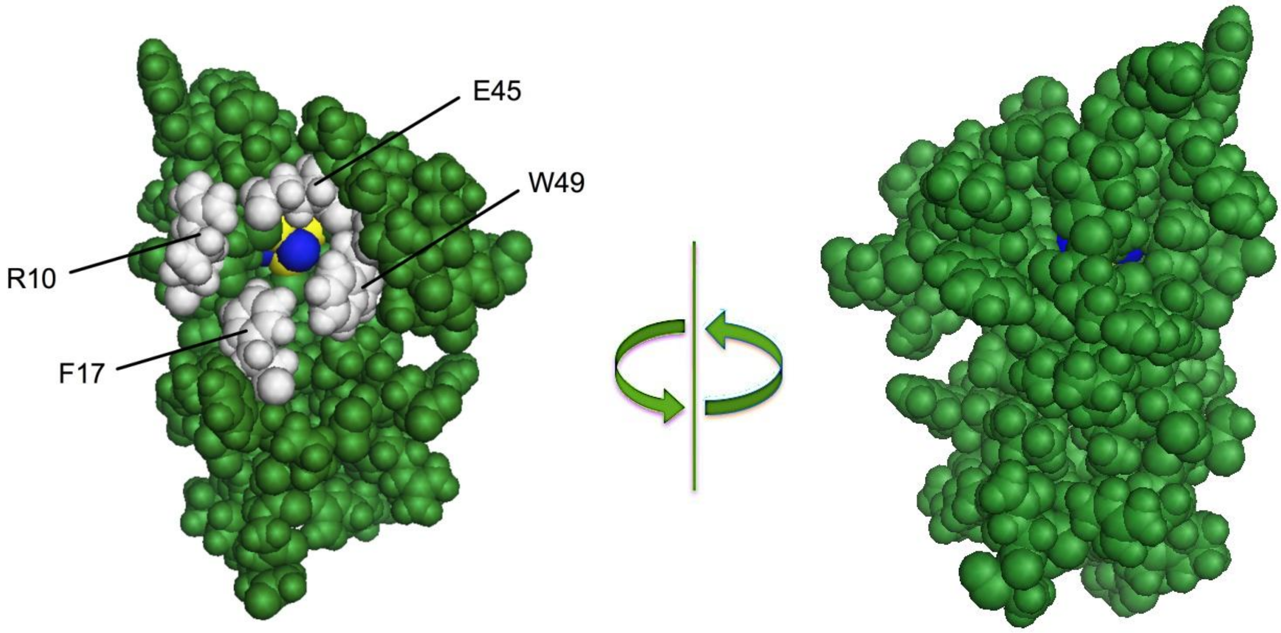

Supplementary Figure 4 | Surface representation of WhiB1 showing solvent access to the iron-sulfur cluster. The WhiB1 surface is shown in green, iron as yellow spheres, sulfur as blue spheres and residues (Arg10, Phe17, Glu45 and Trp49) that form the mouth of the channel leading to the exposed sulfur atom of the cluster are highlighted in gray and single letter amino acid codes. The views are rotated by 180°.

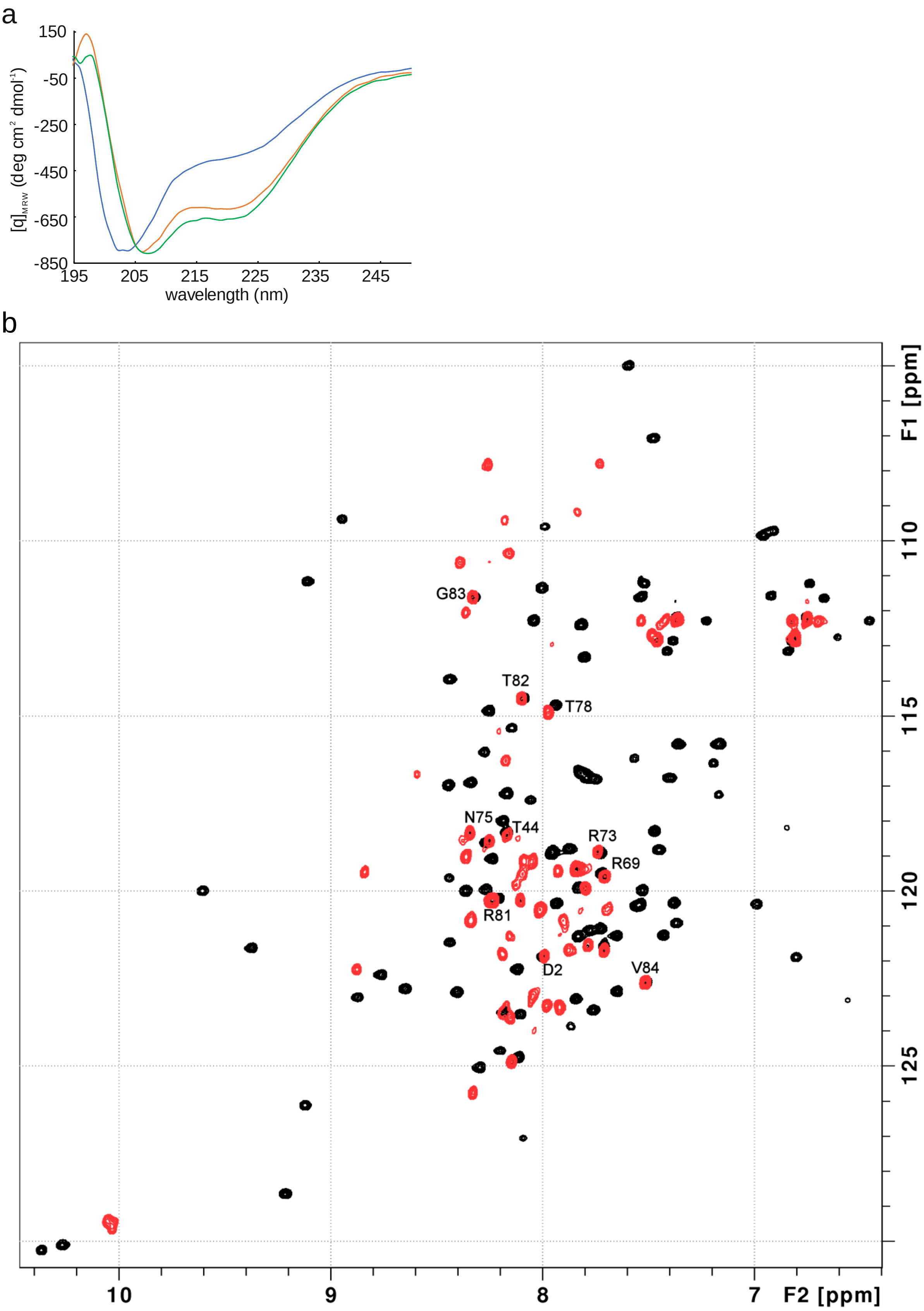

Supplementary Figure 5 | Secondary structure of various forms of WhiB1. **a** Far UV CD spectra of apo- (blue line), holo- (green line) and reconstituted apo- (orange line) WhiB1 (30 µM) in 20 mM NaH_2PO_4 (pH 7.4) containing 0.1 M NaCl at 25°C. **b**, HSQC NMR spectrum of apo-WhiB1 (red) in comparison to holo-protein under the same conditions (black). Amino acids that are found in similar positions in both the holo- and apo-proteins are indicated.

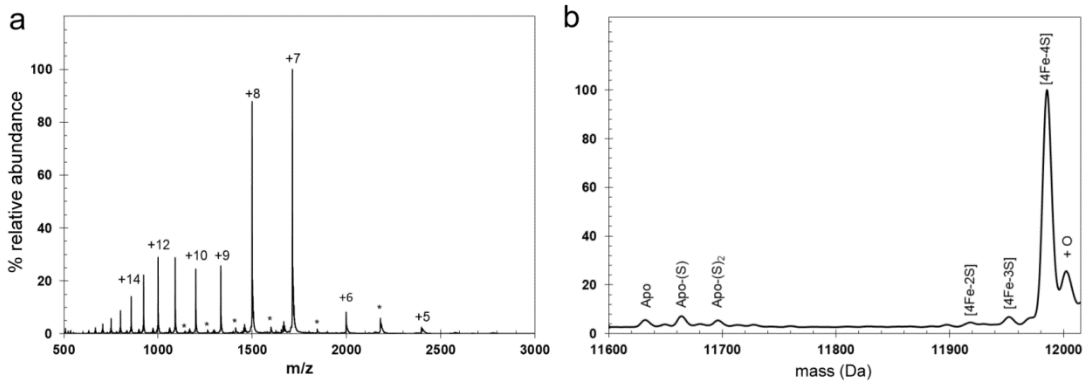

Supplementary Figure 6 | ESI-MS of [4Fe-4S] WhiB1. **a**, m/z spectrum of [4Fe-4S] WhiB1 showing charge states due to monomeric [4Fe-4S] WhiB1. The asterisks indicate charge states due to a minor component of dimeric [4Fe-4S] WhiB1. **b**, Deconvoluted mass spectrum of the monomer region of the WhiB1 sample, revealing the major [4Fe-4S] WhiB1 species along with minor amounts of species that correspond to partially degraded [4Fe-3S] and [4Fe-2S] forms. Small component of apo-WhiB1 and one and two persulfide adducts forms (apo-(S) and apo-$(S)_2$, respectively) are indicated. WhiB1 (20 μM [4Fe-4S]) was in 250 mM ammonium acetate, pH 8.0.

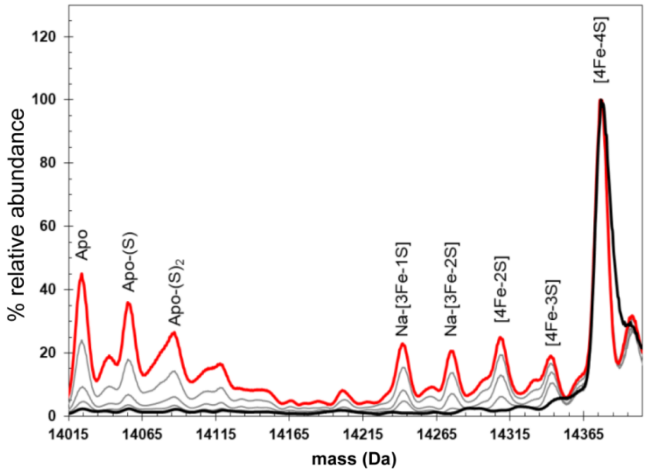

Supplementary Figure 7 | Deconvoluted ESI-MS spectra of *Streptomyces coelicolor* [4Fe-4S] WhiD. The [4Fe-4S] cluster bound form is the only significant species (black spectrum). Application of isCID with increasing energy (up to 100 eV, red spectrum, intermediate energies gave spectra in gray) resulted in detection of cluster fragment species, along with apo-WhiD and sulfur adducts (as indicated) that are very similar to those observed for *M. tuberculosis* WhiB1. WhiD (20 μM [4Fe-4S]) was in 250 mM ammonium acetate, pH 8.0.

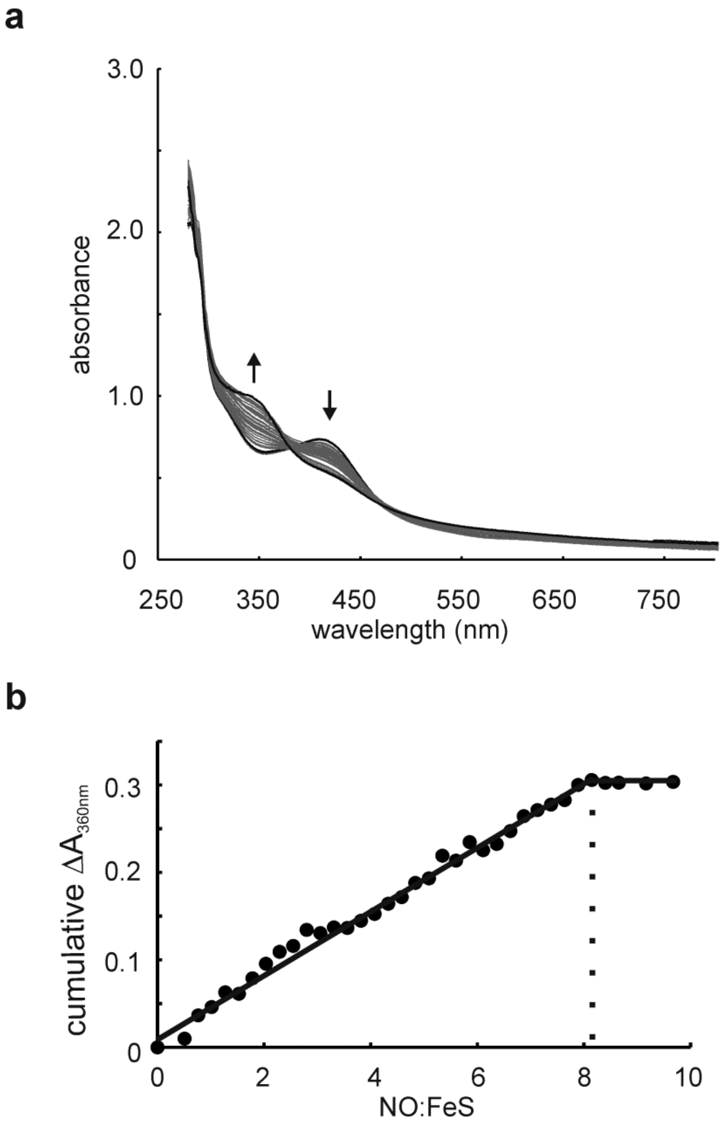

Supplementary Figure 8 | Reaction of the WhiB1 [4Fe-4S] with NO. a, Titration of WhiB1 with NO. Absorbance spectra of $WhiB1_{MS}$ (42 μM cluster) after successive additions of NO up to [NO]/[FeS] of ~10. The arrows indicate the directions of absorbance change. **b**, Changes in absorbance at 360 nm (ΔA_{360nm}) were normalized and plotted against the ratio of NO:FeS. The intersection of the solid lines marked by the dashed line indicates that the reaction is complete upon addition of ~8 NO per [4Fe-4S] cluster.

a

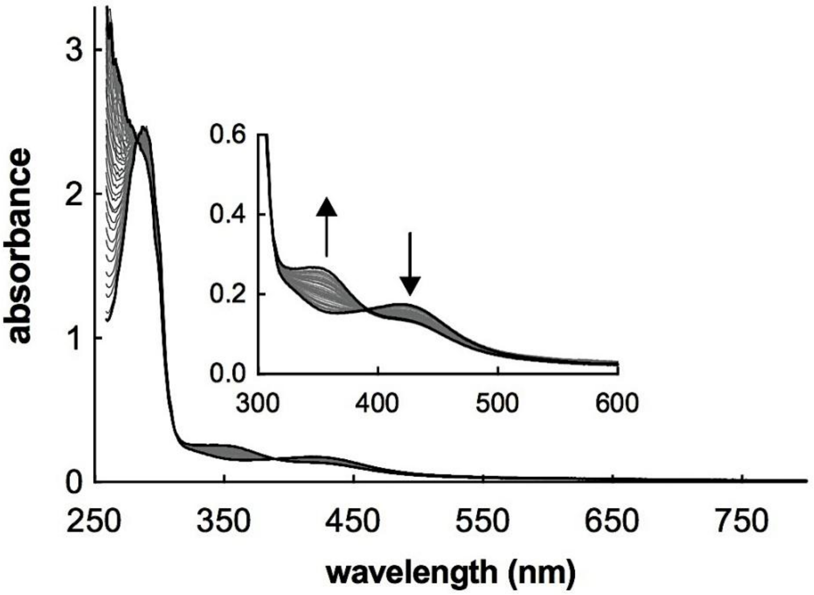

b

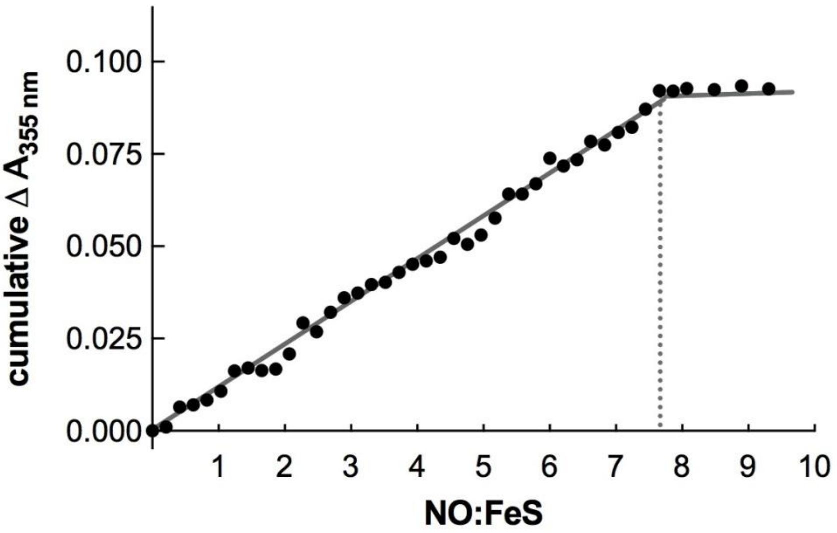

Supplementary Figure 9 | Stoichiometry of the reaction between WhiB1:σ^A complex and NO. a, UV-visible spectra following NO reaction with [4Fe-4S]-WhiB1:σ^A. Increasing amounts (1.05-47.25 μM) of Proli NONOate (equivalent to 2.1-94.5 μM of NO) were introduced to holo-WhiB1:σ^A (10 μM cluster) in 43 additions. The arrows refer to the direction of change in the absorbance. Each spectrum was taken 1.5 min after NO addition. **b**, The cumulative changes in absorbance at 355 nm were plotted versus the NO:[4Fe-4S] ratio. The dashed line indicates the point at which no further change in absorbance was observed.

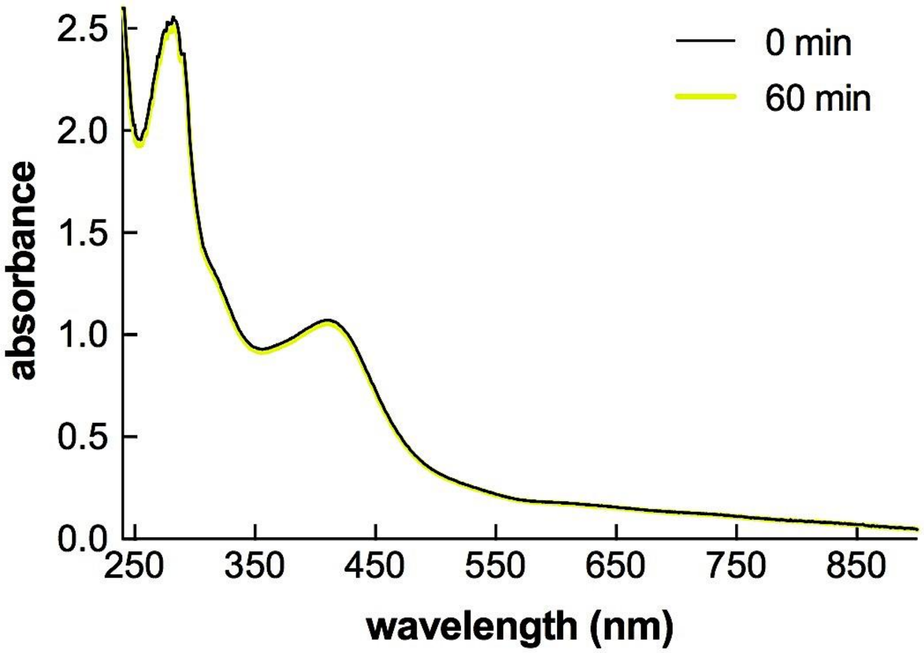

Supplementary Figure 10 | UV-visible spectra of holo-WhiB1:σ^{A}CTD complex. The spectrum of holo-WhiB1:σCTD protein (~65 μM cluster) before (black line) and 60 min after treatment with O_2-saturated buffer (final concentration of 110 μM).

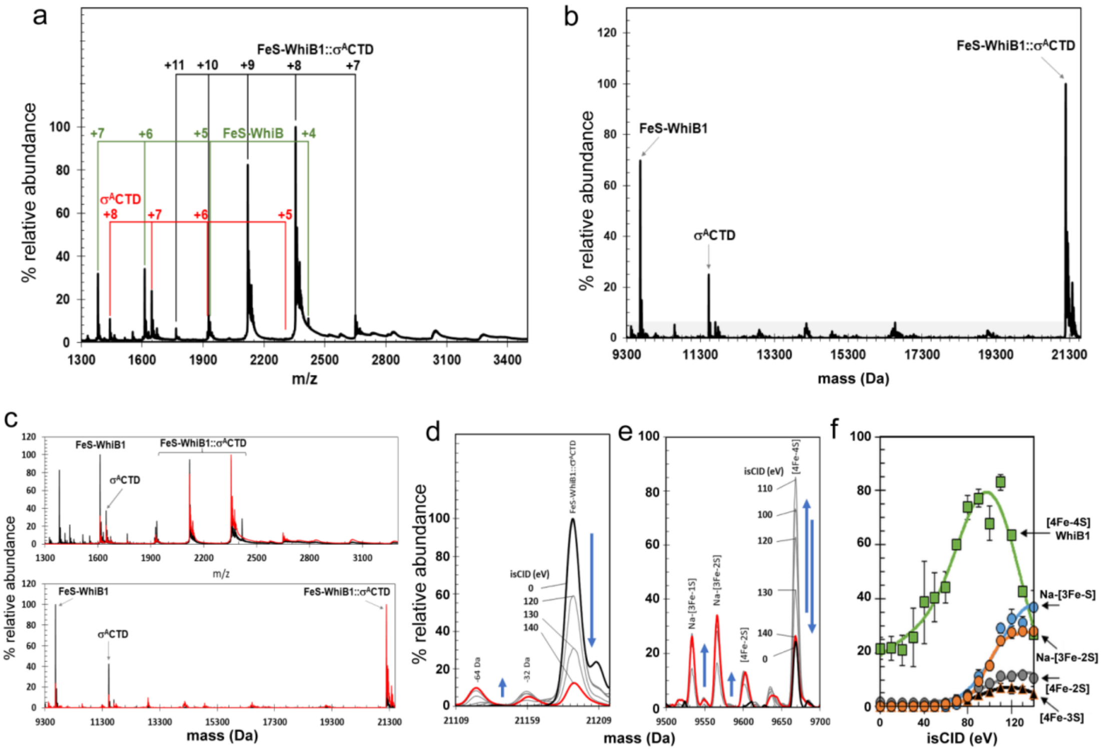

Supplementary Figure 11 | ESI-MS of the WhiB1:σ^{A}CTD complex sample. a, m/z spectrum of the WhiB1:σ^{A}CTD sample showing charge states due to the WhiB1:σ^{A}CTD complex along with those due to non-associated [4Fe-4S] WhiB1 and σ^{A}CTD, as indicated. **b**, Deconvoluted full range mass spectrum, revealing the complex and non-associated [4Fe-4S] WhiB1 and σ^{A}CTD. **c**, m/z (upper panel) and deconvoluted (lower panel) spectra of the WhiB1:σ^{A}CTD sample before (black spectrum) and after (red spectrum) optimization of detection of the WhiB1:σ^{A}CTD complex. **d**, Deconvoluted mass spectra in the WhiB1:σ^{A}CTD complex mass region under optimized conditions with isCID at increasing energies, as indicated. **e**, As in (**d**) but in the dissociated [4Fe-4S] WhiB1 region. Blue arrows indicate the trend of peak intensity changes. **f**, Plots of relative abundance of dissociated WhiB1 species, as indicated, as a function of isCID energy. Note that the data for [4Fe-4S] WhiB1 indicate that it is increasingly dissociated from the complex up to an energy of ~100 eV, above which the cluster begins to degrade. WhiB1:σ^{A}CTD complex (37 μM [4Fe-4S]) was in 250 mM ammonium acetate, pH 8.0.

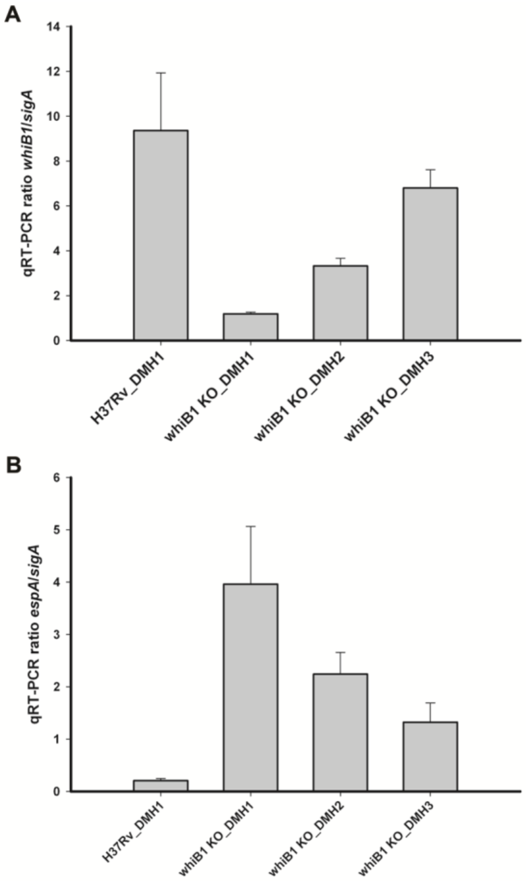

Supplementary Figure 12 | Effect of mutation of *whiB1* on *espA* expression. a, *whiB1* expression was measured by qRT-PCR on RNA from wild type *M. tuberculosis* H37Rv containing the pDHM1 plasmid carrying the *whiB1* gene, and in the *ΔwhiB1* mutant carrying either pDMH1, pDMH2 or pDMH3, as described in Methods. Results were normalized to the *sigA* gene. **b**, *espA* expression was measured using qRT-PCR on the same strains as in Supplementary Fig. 12a. Results were normalized to the *sigA* gene. Data are the means and standard deviations obtained from three biological replicates.

a

F5

1357.CGTCTCACCAAATCACACCGGGCACCGGCCTGAGAAGGGCTTGGGGAGCAGCCAGAGGCGATTGTCGCGGGTGCTGCCGCGCATCATTGATCGG
CCGGCCGGACCAGTCGGGCCTCCCTTGACGTCCGGATCGCACTTCCTGTGCAGCTGGCATGGCTACAGCTCACAGTGACTGCCCCACGATTGCCGGCCA
GGTCCAGTTCAAATTCCGGTGAATTCGCGGACAAAAGCAGCAGGTCAACCAACCGCAGTCAGTCGAGGGTCCCAAACGTGAGCC

F4

CRP Site
AATCGGTGAAATGGCTTGCTGC***AGTGACACCGGTCACA***GGCTTAGCCGACAGCACCGGAATAGCTCAGGCGGGCTATAGAGTCCTA
EspR site C
TAGAAAC***ATTTGCTG***ATAGAATTAACCGCTGTCTTGGCGTGATCTTGATACGGCTCGCCGTGCGACCGGTTGGCTCAGTAGCTGACCACCATGTAACCC
ATCCTCGGCAGGTGTCTACTAAGGCGAGACACCGCATTGGTGGGGCTGCATCGCAAATCGGTCCGAGCATGTAGCACTGCCGTTA

F3

EspR site B
TCCCGGGA***TAGCAAAC***CACCCGGAACCAGGGCTATCCCAGTCGCTCTCCGACGGAGGCCGTTTCGCTTTCCGTTGCCCGATAACTC
MprA site C
CCGAGTGGATATCGGCGTTATCAG***ATTCAGGCTTTTCTTCG***CAAGGTACCGGTGTTCGCTATATTCGGATATCTCGGACGGATAATTACTAAAACTTCA
GTGGTTTAGATAAGGCCGCCGCAATACTTCGCCGATCTTGCCGAGCGCAACGGATTTCCATCGTCGGTTTTCGTCGCCTTATCAA

F2

EspR site A
ACATGATCGGAGATAATGACAGATCGGCCTAGCTAGGTGTTTAGCGGACGCGATTTAGGACAACCGAG***ATTTGCTT***TGCCTCGCAACCATGAGAGCGCC
CCGCTTCGACGCCGAATCGGGTGAGTGATGGTGGGTTAGCACAGCCCTGATTGCGCCACCGGCGAGGTGATTG
MprA site B
TGCCCGCCACGAGGCCGCCGCCGGCTAGCCCCATGAGCACACTATATAGACTCTCCTGCAACAGA***TCTCATACCGATCGAAG***GCGAAGCGCAGGCATC

F1

GACGTCGGAGACACTGCCTTGGGATCGCGCCGCCTACACGGCGGTTGGCGCATTGTCGCAGCGCAGTTGCAGGAGGGCAAATGTGC
MprA site A
GCAGACGATGTAGTCGACAACAAGTGAACATGCCG***TCTTCACGAACTCAAAA***CTGACGATCTGCTTAGCATGAAAAAAACTGTTGACATCGGCCAAGCA
TGACAGCCAGACTGTAGGCCCTACGCGTGCAATGCAGAACCAAGGCTATGCATGGAATCGACGACCGTTGAGATAGGCGGCAGGC.1 ATG *espA*

b

TTAATACGACTCACTATAGGGGAATTGTGAGCGGATAACAATTCCCCTCTAGAAATAATTTTGTTTAAC
TTTAAGAAGGAGATATACATATGGCTAGCATGACTGGTGGACAGCAAATGGGTCGCGGATCCGAATTCG
AGCTCCGTCGACAAGCTTGCGGCCGCACTCGAGCACCACCACCACCACCACTGAGATCCGGCTGCTAAC
AAAGCCCGAAAGGAAGCTGAGTTGGCTGCTGCCACCGCTGAGCAATAACTAGC

Supplementary Figure 13 | The DNA sequence of specific and non-specific competitor. a, The DNA sequence of P*espA* regions, yellow highlighting represents non-labeled forward primer site, red highlighting represents biotin labeled reverse primer sites. MprA, EspR and CRP sites are the binding sites of these regulatory proteins at P*espA*. **b**, The DNA sequence of non-specific competitor, which is not related to the genomic DNA of *M. tuberculosis*.

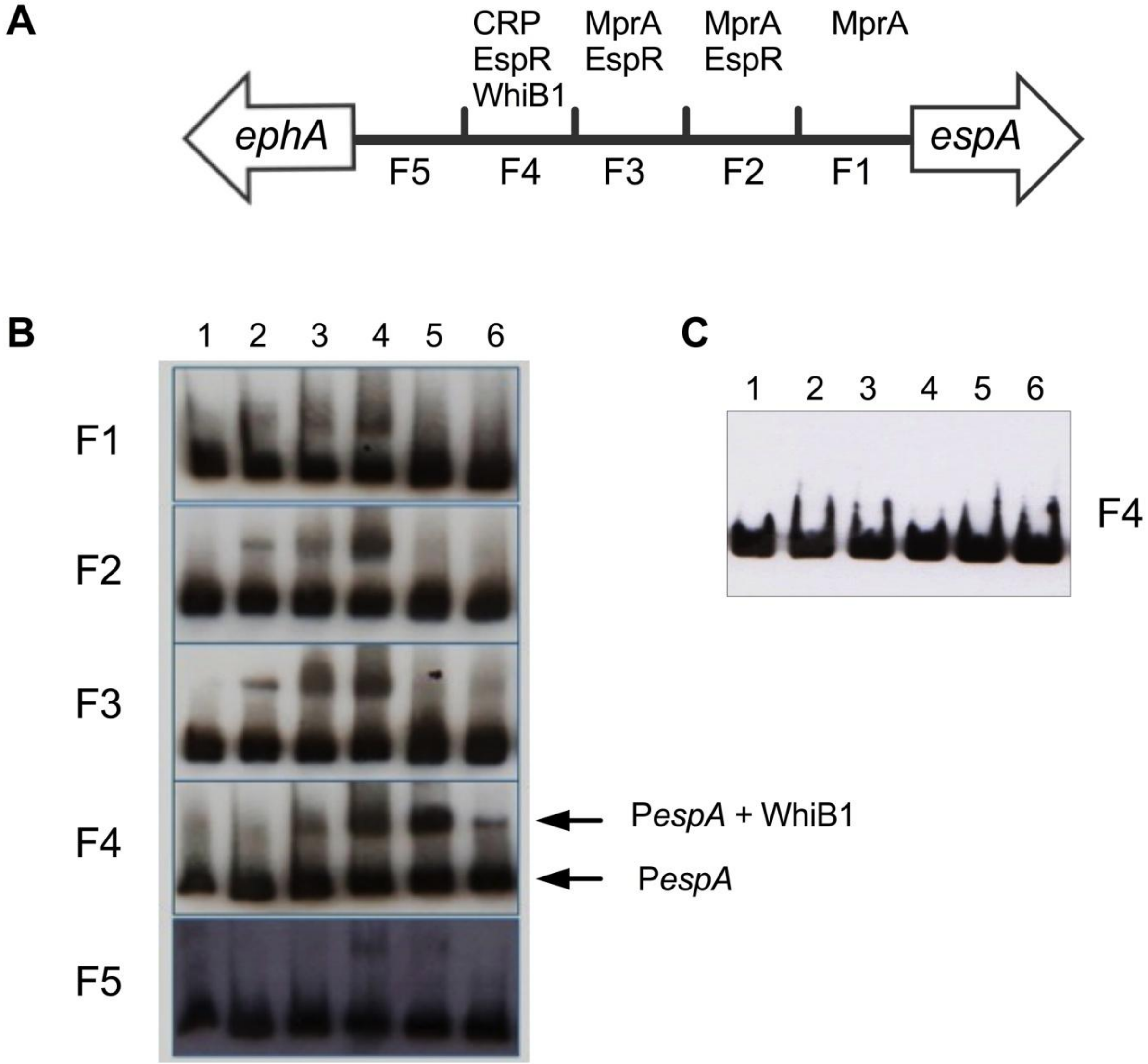

Supplementary Figure 14 | Apo-WhiB1 binding at the *ephA-espA* intergenic region. a, Schematic diagram of the *ephA-espA* intergenic DNA. The binding sites for previously identified regulators of *esp* operon expression (CRP, Rv3676; EspR, Rv3849; MprA, Rv0981) are mapped to the DNA fragments (F1-F5) tested in the electrophoretic mobility shift assays shown in (**b**). **b**, Biotin-labeled DNA fragments (F1 to F5) from the *ephA-espA* intergenic region were incubated with no protein (lane 1); 5, 10, 15 µM apo-WhiB1 (lanes 2 to 4); 15 µM apo-WhiB1 and 200-fold excess of unlabeled non-specific DNA (lane 5); 15 µM apo-WhiB1 and 150-fold excess DNA corresponding to the labeled DNA fragment under test (lanes 6). The locations of the free DNA (P_{espA}) and the WhiB1-DNA complex (P_{espA}+WhiB1) are indicated for DNA fragment F4, which exhibits specific WhiB1 binding. **c**, Holo-WhiB1, which has been previously shown to not bind DNA was incubated with F4[5]; assays are as described for (**b**). WhiB1 was purified after overproduction in *E. coli* JRG6769 (Supplementary Table 3).

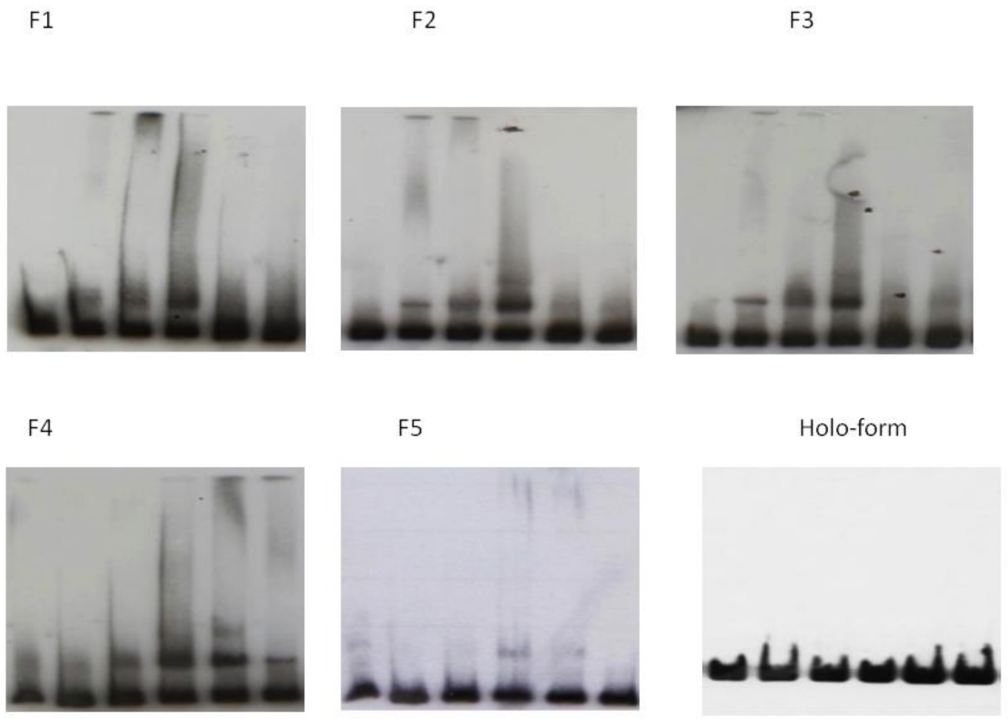

Supplementary Figure 15 | Full gel images for the EMSA data shown in Supplementary Fig. 14.

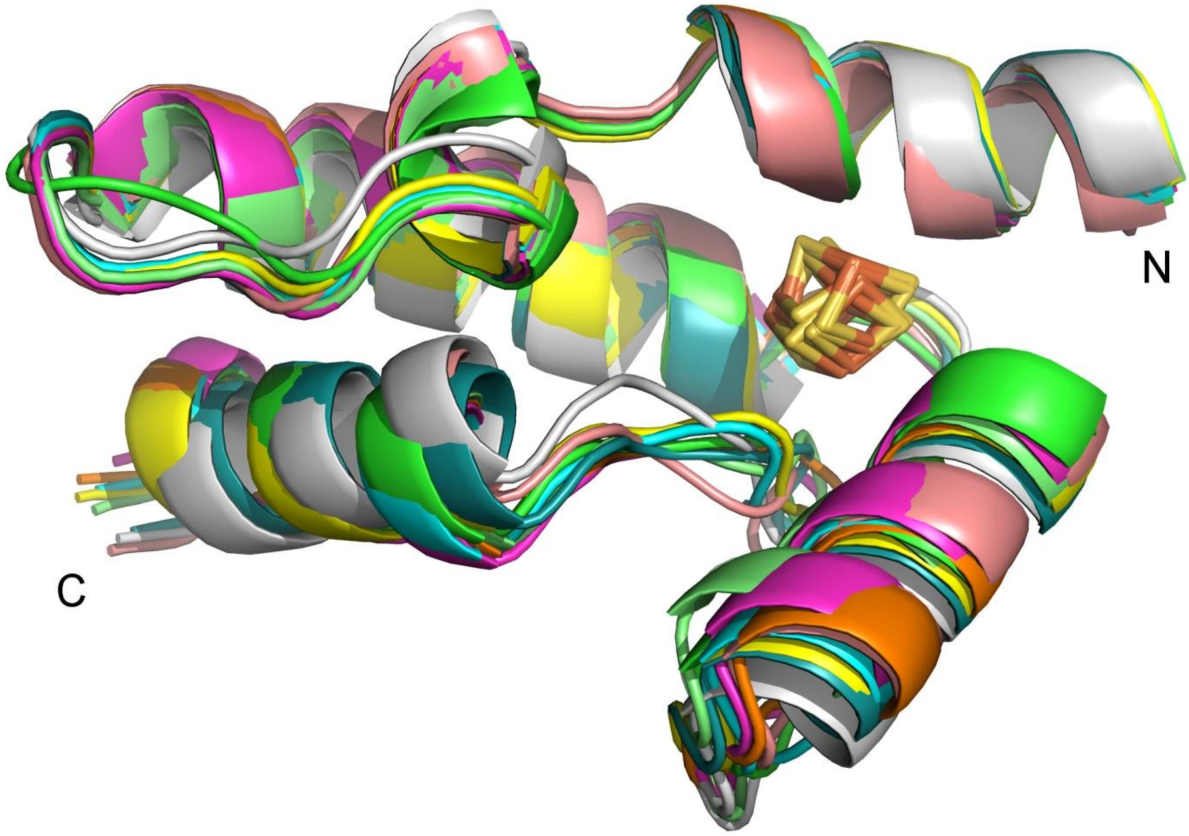

Supplementary Figure 16 | Superimposition of 10 lowest energy NMR structures of WhiB1. The iron-sulfur cluster is shown in brown and yellow, and N- and C-termini are indicated.

Supplementary Table 1 | Predicted and observed masses for WhiB1 and FeS-WhiB1::σ^ACTD species

Species	*Predicted Mass (Da)*[a]	*Average observed Mass (Da)*[b]	*ΔMass (Da)*[c]
WhiB1			
$[4Fe\text{-}4S]^{2+}$	11986	11986	0
Apo	11636	11632	-4
$[4Fe\text{-}3S]^{3+}$ [d]	11953	11953	0
$[4Fe\text{-}2S]^{5+}$ [d]	11919	11919	0
$[3Fe\text{-}2S]^{5+}$-Na [e]	11885	11884	-1
$[3Fe\text{-}S]^{7+}$-Na [e]	11851	11851	0
Apo[d]	11636	11634	-2
Apo-(S^0) [d]	11668	11635	-3
Apo-$(S^0)_2$[d]	11700	11696	-4
FeS-WhiB1::σ^ACTD			
$[4Fe\text{-}4S]^{2+}$ WhiB1::σ^ACTD	21192	21191	-1
$[4Fe\text{-}3S]^{3+}$ WhiB1::σ^ACTD [d]	21159	21159	0
$[4Fe\text{-}2S]^{5+}$ WhiB1::σ^ACTD [d]	21125	21124	-1
σ^ACTD	11523	11522	-1
WhiB1			
$[4Fe\text{-}4S]^{2+}$	9669	9668	-1
$[4Fe\text{-}3S]^{3+}$ [e]	9636	9635	-1
$[4Fe\text{-}2S]^{5+}$ [e]	9602	9603	+1
$[3Fe\text{-}2S]^{5+}$-Na [e]	9568	9566	-2
$[3Fe\text{-}S]^{7+}$-Na [e]	9534	9533	-1
Apo	9319	9315	-4
Apo-(S^0) [e]	9351	9348	-3
Apo-$(S^0)_2$ [e]	9383	9381	-2

[a]The predicted mass depends on the cluster/cluster fragment charge because binding is assumed to be charge compensated[1]. Cluster charge states are as observed previously[1].
[b]The average observed mass is derived from at least four independent experiments, with standard deviation of ± 1 Da.
[c]The difference between the average observed and predicted masses.
[d]Significantly enhanced by isCID
[e]Generated via isCID

Supplementary Table 2 | Predicted and observed masses for WhiD species

Species	*Predicted Mass (Da)[a]*	*Average observed Mass (Da)[b]*	*ΔMass (Da)[c]*
WhiD			
Apo	14158	-	-
(ΔMet) WhiD			
$[4Fe\text{-}4S]^{2+}$	14377	14377	0
Apo	14027	14025	-2
$[4Fe\text{-}3S]^{3+}$ [d]	14344	14344	0
$[4Fe\text{-}2S]^{5+}$ [d]	14310	14309	-1
$[3Fe\text{-}2S]^{5+}$-Na [d]	14277	14276	-1
$[3Fe\text{-}S]^{7+}$-Na [d]	14243	14243	0
Apo [d]	14027	14024	-3
Apo -(S^0) [d]	14059	14056	-3
Apo-$(S^0)_2$ [d]	14091	14087	-4

[a]The difference in predicted mass depending on the overall charge on the cluster is due to charge compensation of cluster binding. A lower overall cluster charge would result in more protons remaining bound and consequently a higher mass[1].
[b]The average observed mass is derived from at least four independent experiments, with standard deviation of ± 1 Da.
[c]The difference between the average observed and predicted masses.
[d]Generated via isCID

Supplementary Table 3 | Bacterial strains and plasmids used in this study

Bacterial strain	*Relevant characteristics*	*Reference or source*
Escherichia coli BL21(DE3)	Host for protein production after transformation with pET-based expression plasmids	Laboratory stock
E. coli BTH101	Host for bacterial two-hybrid analysis; *cya* mutant	2
E. coli JRG5386	*E. coli* BTH101 pGS1670 pGS1672	This work
E. coli JRG5387	*E. coli* BTH101 pGS1671 pGS1673	This work
E. coli JRG6769	*E. coli* BL21(DE3) pGS2500	This work
E. coli JRG6859	*E. coli* BL21(DE3) pGS2183 pGS2560	This work
E. coli JRG6862	*E. coli* BTH101 pGS2567 pGS2568	This work
Mycobacterium smegmatis mc^2 155	Host for *M. tuberculosis* WhiB1 protein production	3
M. smegmatis mc^2 155 JRG6798	*M. smegmatis* mc^2 155 pGS2524	This work
Mycobacterium tuberculosis H37Rv	Wild-type virulent strain	4
M. tuberculosis H37Rv *ΔwhiB1*/pDMH1	Complemented *whiB1* deletion mutant	5
M. tuberculosis H37Rv *ΔwhiB1*/pDMH2	Complemented *whiB1* deletion mutant	5
M. tuberculosis H37Rv *ΔwhiB1*/pDMH3	Complemented *whiB1* deletion mutant	5
Plasmids	*Relevant characteristics*	*Reference or source*
pET21a	Vector for protein overproduction in *E. coli* BL21(DE3)	Novagen
pET28a	Vector for overproduction of *N*-terminally His_6-tagged proteins in *E. coli* BL21(DE3)	Novagen
pDMH1	*whiB1* complementing derivative of pKP203 (293 bp 5'–14 bp 3')	5
pDMH2	*whiB1* complementing derivative of pKP186 (1192 bp 5'–14 bp 3')	5
pDMH3	*whiB1* complementing derivative of pKP186 (1192 bp 5'–535 bp 3')	5
pGS1669	pUT18; codes for the T18 fragment (amino acids 225 to 399) of *Bordetella pertussis* adenylate cyclase; Ap^R	2
pGS1670	pUT18c; codes for the T18 fragment (amino acids 1 to 244) of *B. pertussis* adenylate cyclase; Ap^R	2
pGS1671	pUT18c-zip; codes for the leucine zipper from GCN4 fused to T18; Ap^R	2
pGS1672	pKT25; codes for the T25 fragment (amino acids 1 to 244) of *B. pertussis* adenylate cyclase; Ap^R	2
pGS1673	pKT25-zip; codes for the leucine zipper from GCN4 fused to T25; Kn^R	2
pGS2183	pET28a derivative for expression of *M. tuberculosis sigA* in *E. coli* BL21(DE3); Kn^R	This work
pGS2314	pRLG770 derivative containing the *espA* promoter region (1119 bp upstream to 42 bp downstream of *espA* ATG); Ap^R	This work
pGS2500	pET28a derivative for expression of	This work

	His_6-thrombin cleavage site-tagged *M. tuberculosis whiB1* in *E. coli* BL21(DE3); Kn^R	
pGS2524	pMyNT derivative for expression of His_6-TEV cleavage site-tagged *M. tuberculosis whiB1* in *M. smegmatis*; Hyg^R	This work
pGS2560	pET21a derivative for expression of untagged *M. tuberculosis whiB1* in *E. coli* BL21(DE3); Ap^R	This work
pGS2563	pET21a derivative foe expression of untagged *M. tuberculosis whiB1*-Cys40Ala in *E. coli* BL21(DE3); Ap^R	This work
pGS2566	pET28a derivative for expression of His6-tagged *C*-terminal domain (amino acids Ala447 to Asp528 of *M. tuberculosis sigA* in *E. coli* BL21(DE3); Kn^R	This work
pGS2567	pUT18 derivative coding for a T18 σ^ACTD fusion; Ap^R	This work
pGS2568	pUT25 derivative coding for a T25 WhiB1 fusion; Kn^R	This work
pMyNT	Mycobacterial expression plasmid; Hyg^R	Gift from Annabel Parret & Matthias Wilmanns (Addgene plasmid # 42191)

Supplementary Table 4 | Oligonucleotides used in this study

Oligonucleotide	*Sequence*	*Purpose*
ECWb1F	ATATATCATATGGATTGGCGCCACA AGGCG	Amplification of *whiB1* for insertion into pET28a
ECWb1R	ATATATGTCGACTCAGACCCCGGTACGGGC TTT	Amplification of *whiB1* for insertion into pET28a
MSWb1F	TATATACCATGGATTGGCGCCACAAGGCG	Amplification of *whiB1* for insertion into pMyNT
MSWb1R	TATATAAAGCTTTCAGACCCCGGTACGGGCTTT	Amplification of *whiB1* for insertion into pMyNT
pUT18-SigAF	ATATATAAGCTTGATGGCCGTCGACGCGGTGTCC	Amplification of σ^{A}CTD for insertion into pUT18
pUT18-SigAR	ATATATGGTACCCGGTCCAGGTAGTCGCGCAGGAC	Amplification of σ^{A}CTD for insertion into pUT18
pKT25-WB1F	TATATATCTAGAGATGGATTGGCGCCACAAGGCG	Amplification of *whiB1* for insertion into pKT25
pKT25-WB1R	ATATATGGTACCTCAGACCCCGGTACGGGCTTT	Amplification of *whiB1* for insertion into pKT25
SigAc82F	ATATATCATATGGCCGTCGACGCGGTGTCCTTCA	Amplification of σ^{A}CTD for insertion into pET28a
SigACTDR	ATATATGTCGACTCAGTCCAGGTAGTCGCGCAGG	Amplification of σ^{A}CTD for insertion into pET28a
espAF	CCGGGTGATGGCTGGTTAGG	qRT-PCR
espAR	AGGCTGATGAGCTGACGAT	qRT-PCR
whiB1F	TTCTTCCCGGTAGGAAACAGTG	qRT-PCR
whiB1R	ATTACAGACCAGTTCGCGTCA	qRT-PCR
sigAF	CTCGACGCTGAACCAGACCT	qRT-PCR
sigAR	AGGTCTTCGTGGTCTTCGT	qRT-PCR

Supplementary Table 5 | NMR and refinement statistics for WhiB1

	Value
**NMR distance and dihedral constraints**	
Distance constraints[a]	
Total	218
Intra-residue	10
Inter-residue	
Sequential ($\|i – j\|$ = 1)	120
Medium-range ($\|i – j\| \leq 4$)	36
Long-range ($\|i – j\| > 4$)	20
Upper bounds to cluster	19
Lower bounds to cluster	13
Hydrogen bonds[b]	34
Total dihedral angle restraints[c]	
ϕ	67
ψ	63
χ_1	28
**Structure statistics**[d]	
Violations (mean and s.d.)	
Distance constraints (Å)	0.091 ± 0.076
Dihedral angle constraints (º)	0.39 ± 0.76
Max. dihedral angle violation (º)	5.35
Max. distance constraint violation (Å)	0.42
Deviations from idealized geometry	
Bond lengths (Å)	0.0032 ± 0
Bond angles (º)	0.486 ± 0.018
Impropers (º)	0.0449 ± 0.009
Average pairwise r.m.s. deviation (Å)	
Heavy	2.08
Backbone	0.92

[a]NOE cross-peak intensities were divided into three groups with upper bounds of 3.0, 4.0 and 5.0 Å.
[b]Defined by helices 1-12, 25-38, 43-53, 64-76.
[c]Implemented using a range of ±1 standard deviation derived from TALOS-N.
[d]Structure statistics were calculated for the 10 lowest energy refined structures (Supplementary Fig. 16) from a total of 60 calculated structures.

Supplementary References

1. Johnson, K. A. *et al*. Probing the stoichiometry and oxidation states of metal centers in iron-sulfur proteins using electrospray FTICR mass spectrometry. *Anal. Chem.* **72**, 1410-1418 (2000).

2. Karimova, G., Pidoux, J., Ullmann, A. & Ladant, D. A bacterial two-hybrid system based on a reconstituted signal transduction pathway. *Proc. Natl. Acad. Sci. USA* **95**, 5752–5756 (1998).

3. Snapper, S. B. *et al*. Isolation and characterization of efficient plasmid transformation mutants of *Mycobacterium smegmatis*. *Mol. Microbiol.* **4**, 1911-1919 (1990).

4. Kubica, G. P., Kim, T. H. & Dunbar, F. P. Designation of strain H37Rv as the neotype of *Mycobacterium tuberculosis*. *Int. J. Syst. Bacteriol.* **22**, 99-106 (1972).

5. Smith, L. J. *et al*. *Mycobacterium tuberculosis* WhiB1 is an essential DNA-binding protein with a nitric oxide-sensitive iron-sulfur cluster. *Biochem. J.* **432**, 417-427 (2010).
